# The first complete mitochondrial genome sequence of *Cacopsylla citrisuga* (Yang & Li), a new insect vector of Huanglongbing in Yunnan Province, China

**DOI:** 10.1080/23802359.2021.1875908

**Published:** 2021-02-14

**Authors:** Yanjing Wang, Yijing Cen, Yurong He, Yixuan Wu, Sunbin Huang, Jinming Lu

**Affiliations:** a College of Agriculture and Food Sciences, Zhejiang Agriculture and Forestry University, Hangzhou, Zhejiang, China; b Department of Entomology/ Key Laboratory of Bio-Pesticede Innovation and Application, College of Plant Protection, South China Agricultural University, Guangzhou, Guangdong, China; c Mécanismes adaptatifs et évolution (MECADEV), UMR 7179 CNRS-MNHN, Muséum National d’Histoire Naturelle, CP50, 57 rue Cuvier, Paris, France; d College of Forestry and Biotechnology, Zhejiang Agriculture and Forestry University, Hangzhou, Zhejiang, China

**Keywords:** *Cacopsylla citrisuga*, mitochondrial genome, phylogenetic analysis

## Abstract

*Cacopsylla citrisuga* (Yang & Li) is an important pest-threatening *Citrus* and *Poncirus* plants (Rutaceae) and a newly identified insect vector of citrus Huanglongbing. The complete mitochondrial genome of *C. citrisuga* was 14,906 bp in length, with 37 genes, including 13 protein-coding genes (PCGs), 22 transfer RNA genes (tRNAs), two ribosomal RNA genes (rRNAs). The phylogenetic trees inferred from Bayesian inference and maximum likelihood analyses confirmed *C. citrisuga* as a member of the genus *Cacopsylla*. Our phylogenetic analyses suggested that the *Cacopsylla* is paraphyletic, and confirmed *C. citrisuga* as a member of clade-I under *Cacopsylla*. The complete mitochondrial genome of *C. citrisuga* will provide important information for the phylogeny and evolution analysis of *Cacopsylla*.

Huanglongbing (HLB), known as citrus greening disease, is associated with phloem-restricted alpha-proteobacterium: ‘*Candidatus* Liberibacter asiaticus,’ ‘*Ca.* L. africanus,’ and ‘*Ca.* L. americanus’ (Bové 2006). *Cacopsylla citrisuga* (Yang & Li) (Hemiptera: Psyllidae), is an important pest of *Citrus* and *Poncirus* plants (Rutaceae), has been proved to be a new insect vector of ‘*Candidatus* Liberibacter asiaticus’ (Cen et al. 2012). This pest belongs to the genus *Cacopsylla* Ossiannilsson, 1970, which is the largest psyllid genus comprising *ca.* 470 species (https://hemiptera-databases.org/). To date, 359 complete or near complete mitogenomes from species of the psyllids (family Psylloidea) have been sequenced, among which only 24 are from the genus *Cacopsylla*; in addition, the evolution relationship of *Cacopsylla citrisuga* with other psyllids is still less described. In this study, we analyzed and reported the first mitogenomes of *Cacopsylla citrisuga* and built its phylogenetic history with other psyllids.

The adult specimens used in this study were collected at Mengxiu, Yunnan, China (24.075618°N, 97.741441°E) in June 2018. The collected specimen was kept in 80% ethyl alcohol and deposited in Insect Collection of Zhejiang A&F University (ZAFU-INSECT-Y501). Whole genome DNA was extracted using the QIAamp DNA Micro Kit (Qiagen Hilden, Germany) and sequenced on a HiSeq 2000 platform (Illumina, USA) with a paired-end 150-bp sequencing strategy. Clean data screening and *de novo* assembly also follows the method of Wang et al. (2018). We obtained 14,906 base-pair of assembled, circular mitochondrial genomic sequences for *C. citrisuga* (GenBank accession number: MT990978). The assembled psyllid mitochondrial genome fit well within the conserved nature of the mitochondrial genomes of Hemiptera (Wang et al. 2015): contain 13 protein-encoding genes (PCGs; *COI*-*III*, *ND1*-*6*, *ND4L*, *ATP6*, *ATP8*, and *CytB*), 22 tRNA genes, two ribosomal RNAs and a non-coding A + T-rich region (Supplementary Table S1; Figure S1). The overall base composition of the mitochondrial genome is A (38.2%), T (34.5%), C (17.7%), G (9.6%).

Phylogenetic trees were constructed based on 13 PCGs sequences from 40 species (Supplementary Table S2) by Bayesian Inference (BI) and maximum likelihood (ML) analyses. The 40 mitochondrial sequences were aligned by the MAFFT version 7 software (Katoh and Standley 2013). Bayesian Inference was conducted by MrBayes 3.2.1 (Ronquist and Huelsenbeck 2003) and maximum likelihood (ML) analyses implemented in IQ-TREE 1.5.5 (Nguyen et al. 2015). *Cacopsylla* was recovered as paraphyletic clades in the present study, consistently with previous paraphyletic research (Percy et al. 2018; Cho et al. 2019). Both BI and ML analyses strongly supported *C. citrisuga* as a member of *Cacopsylla*, and *C. citrisuga* was embraced in a clade consisting *C. coccinea*, *C. ambigua*, *C. bruneipennis*, *C. spiculata*, *C. saliceti*, *C.* sp. L2, *C.* sp. 4, *C.* sp., *C. magnicauda*, *C. stricklandi*, *C. pyricola*, *C. notata*, *C. pyri*, with strong supports (PP ≥ 0.90; BP ≥ 95; Figure 1). The availability of this additional genetic information promises to open the way for studies of phylogeny of *Cacopsylla*. This report is the first molecular characterization of complete mitogenome of the important agricultural pest, *C. citrisuga*. The availability of this additional genetic information will be useful for further investigating the evolutionary relationships within *Cacopsylla*.

**Figure 1.**
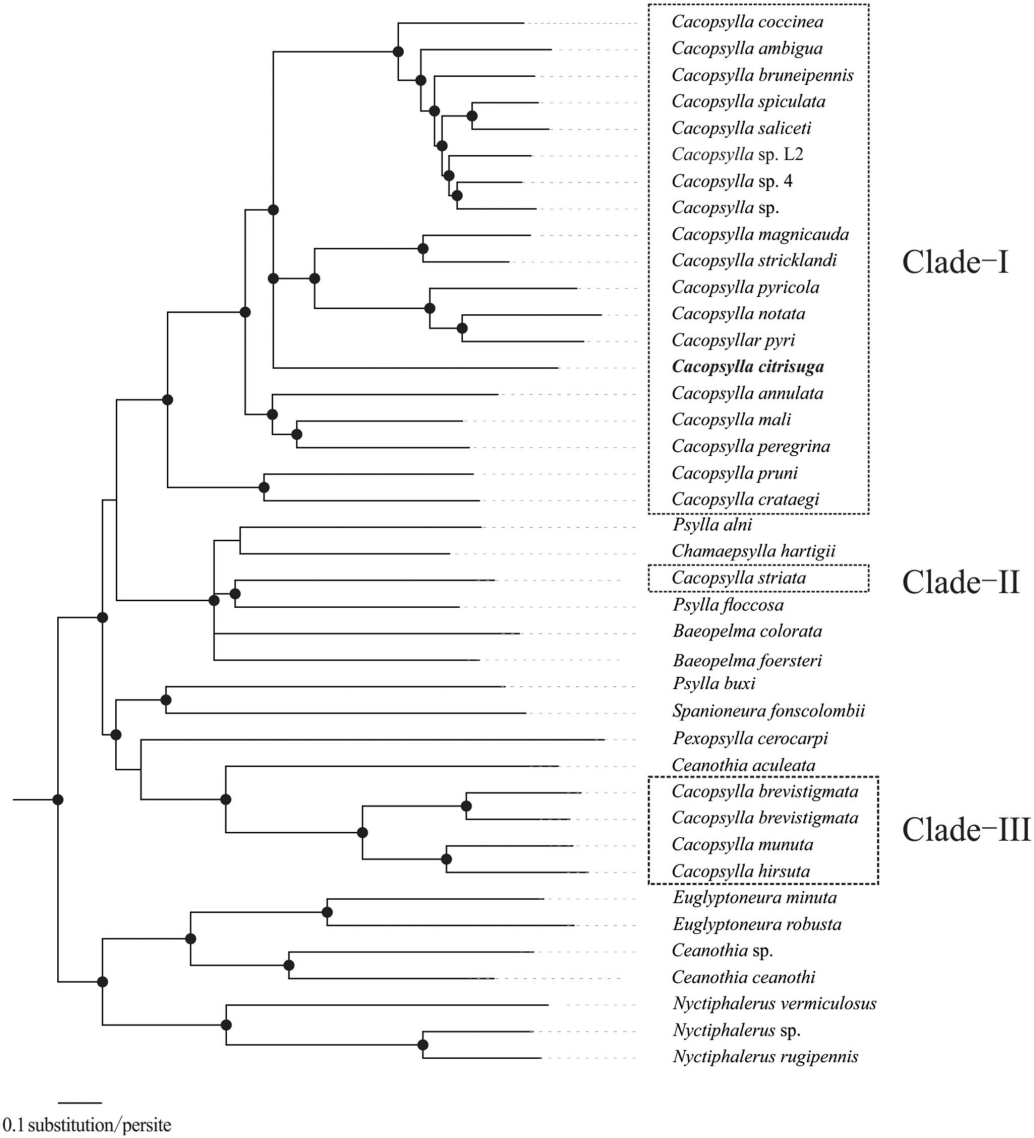
Bayesian phylogenetic inferred from 40 psyllids mitogenome sequences based on 13 PCGs. The node support values indicate the Bayesian posterior probabilities (PP) and the bootstrap (BP) values. Cycles on the nodes indicated BI posterior probabilities ≥ 0.90 and ML bootstrap values ≥ 95 for clades.

## Data Availability

The data that support the findings of this study are openly available in GenBank of NCBI at https://www.ncbi.nlm.nih.gov, reference number MT990978
